# Supplementary material for: CaV1.1 voltage-sensing domain III exclusively controls skeletal muscle excitation-contraction coupling
Source: Nat Commun. 2024 Aug 28;15:7440. doi: 10.1038/s41467-024-51809-5 (PMC11358481; doi:10.1038/s41467-024-51809-5)
Supplement: Supplementary file 1 — Supplementary Information [file 41467_2024_51809_MOESM1_ESM.pdf]

Supplementary Table 1. Biophysical properties of calcium currents and depolarization-induced calcium transients in dysgenic myotubes reconstituted with different $Ca_V1.1$ channel constructs.

ICa, current properties

VSD	Currents	*N*	*n*	ICa (pA/pF)	P-value	% of control	$V_{0.5}$ (mV)	P-value	diff. (mV)	TTP (ms)	P-value	diff. (ms)
VSD I	Ca_V1.1a	4	10	-1.6 ± 0.2			32.3 ± 1.9			156 ± 18.5		
VSD I	Ca_V1.1a_VSDI-R4A	5	9	NO CURRENTS								
VSD I	Ca_V1.1e	6	10	-10.1 ± 1.9	0,01	0,4	5.2 ± 1.3	<0.0001	+22.9	90.6 ± 17.7	0,34	25,2
VSD I	Ca_V1.1e_VSDI-R4A	4	9	-4 ± 0.5			28.1± 1.3			115.7± 18.5		
VSD II	Ca_V1.1a	3	9	-1.1 ± 0.2	0,003	2,3	29.3 ± 3.1	0,2	-4.8	111.1 ± 18.5	0,002	203,3
VSD II	Ca_V1.1a_VSDII-K4A	4	7	-2.5 ± 0.4			24.6 ± 1.4			314.4 ± 54.0		
VSD II	Ca_V1.1e	9	12	-6.2 ± 0.5	0,037	1,3	3.8 ± 2.2	0,006	-12.5	139.7 ± 15.2	0,34	35,8
VSD II	Ca_V1.1e_VSDII-K4A	4	6	-8.1 ± 1.6			-8.7 ± 3.5			175.5 ± 42.2		
VSD II	Ca_V1.1e	7	10	-10.5 ± 1.7	0,32	0,8	2.4 ± 2.6	0,035	-7.8	61.8 ± 15.4	0,21	27,4
VSD II	Ca_V1.1e_VSDII-L_{IV}	7	10	-8.3 ± 1.3			-5.4 ± 1.3			89.2 ± 14.2		
VSD III	Ca_V1.1a	3	9	-1.1 ± 0.1			32.1 ± 2.0			131.6 ± 17.6		
VSD III	Ca_V1.1a_VSDIII-R4A	4	11	NO CURRENTS								
VSD III	Ca_V1.1e	9	12	-6.2 ± 0.5	0,01	0,4	3.8 ± 2.2	0,001	+17.7	139.7 ± 15.2	0,27	31,3
VSD III	Ca_V1.1e_VSDIII-R4A	4	5	-2.2 ± 0.5			21.5 ± 3.7			108.4 ± 20.5		
VSD III	Ca_V1.1e	7	9	-5.8 ± 0.5	0,5	0,9	5.6 ± 1.1	0,51	-1,2	85.7 ± 15.7	0,16	27,2
VSD III	Ca_V1.1e_VSDIII-L_{IV}	6	11	-5.3 ± 0.5			4.4 ± 1.3			112.9 ± 11		
VSD IV	Ca_V1.1a	7	9	-1.2 ± 0.1	0,9	1,00	27.2 ± 1.7	0,04	-5,8	126.4 ± 14.9	0,3	17,4
VSD IV	Ca_V1.1a_VSDIV-K4A	6	9	-1.2 ± 0.2			21.4 ± 1.9			108.8 ± 9		
VSD IV	Ca_V1.1e	6	15	-9.5 ± 1.1	0,002	0,5	7.1 ± 1.2	0,39	+1.4	81.4 ± 8.5	0,5	8,2
VSD IV	Ca_V1.1e_VSDIV-K4A	6	15	-4.9 ± 0.7			8.2 ± 1.0			89.5 ± 7.7		

ECC, calcium transients

VSD	ECC	*N*	*n*	Df/F0	P-value	% of control	$V_{0.5}$ (mV)	P-value	diff. (mV)
VSD I	Ca_V1.1a	4	10	0.5 ± 0.1	0,96	1,00	1.7 ± 2.2	0,62	+1.3
VSD I	Ca_V1.1a_VSDI-R4A	5	9	0.5 ± 0.2			3 ± 0.8		
VSD I	Ca_V1.1e	6	10	0.6 ± 0.2	0,49	0,7	2.3 ± 1.7	0,84	+0.5
VSD I	Ca_V1.1e_VSDI-R4A	4	9	0.4 ± 0.1			2.8± 1.6		
VSD II	Ca_V1.1a	3	9	2.6 ± 0.4	0,44	0,8	5 ± 2.9	0,69	-1.5
VSD II	Ca_V1.1a_ VSDII-K4A	4	7	2.1 ± 0.2			3.5 ± 1.6		
VSD II	Ca_V1.1e	9	12	1.9 ± 0.4	0,82	1,1	-2.7 ± 2.3	0,011	-11.5
VSD II	Ca_V1.1e_ VSDII-K4A	4	6	2.1 ± 0.8			-14.2 ± 3.1		
VSD II	Ca_V1.1e	7	10	1.5 ± 0.3	0,17	0,7	-6.2 ± 2.0	0,049	-6.3
VSD II	Ca_V1.1e_VSDII-L_{IV}	7	10	1 ± 0.2			-12.5 ± 2.3		
VSD II	Ca_V1.1nc	9	11	1.2 ± 0.2	0,76	0,9	-1.0 ± 2.6	0,97	+0.1
VSD II	Ca_V1.1nc_VSDII-L_{IV}	5	8	1.1± 0.4			-0.9 ± 2.9		
VSD III	Ca_V1.1a	3	9	1.9 ± 0.5	0,077	0,4	2.2 ± 2.2	<0.0001	+44.8
VSD III	Ca_V1.1a_VSDIII-R4A	4	11	0.8 ± 0.5			47.4 ± 5.2		
VSD III	Ca_V1.1e	9	12	1.9 ± 0.4	0,025	0,2	-2.7 ± 2.3	<0.0001 (both)	+22.9
VSD III	Ca_V1.1e_VSDIII-R4A (1st)	4	5	X			20.1 ± 3.2		
VSD III	Ca_V1.1e_VSDIII-R4A (2nd)	4	5	0.4 ± 0.1			97.2 ± 4.1		+99.9
VSD III	Ca_V1.1e	7	9	1.4 ± 0.2	0,4	0,8	-2.0 ± 1.3	<0.0001 (both)	-59,9
VSD III	Ca_V1.1e_VSDIII-L_{IV} (1st)	6	11	1.1 ± 0.2			-61.9 ± 1.3		
VSD III	CaV1.1e_VSDIII-L_{IV} (2nd)	6	11	X			-14.4 ± 2.0		-12,4
VSD III	Ca_V1.1nc	6	11	1.2 ± 0.4	0,2	0,5	-0.9 ± 1.1	<0.0001	-61.8
VSD III	Ca_V1.1nc_VSDIII-L_{IV}	4	9	0.6 ± 0.1			-62.8 ± 1.2		
VSD IV	Ca_V1.1a	7	9	1.7 ± 0.3	0,3	0,7	10.7 ± 2.6	0,19	+4.2
VSD IV	Ca_V1.1a_VSDIV-K4A	6	9	1.2 ± 0.2			14.8 ± 1.7		
VSD IV	Ca_V1.1e	6	15	1.5 ± 0.3	0,44	0,8	4.5 ± 2.2	0,42	+2.3
VSD IV	Ca_V1.1e_VSDIV-K4A	6	15	1.2 ± 0.3			6.8 ± 1.9		

Ca_V1.1a = adult isoform
Ca_V1.1e = embryonic isoform
Ca_V1.1nc = non-conducting

N = number of independent transfections
n = number of recorded cells

Statistics: Mean ± SEM; mutants and wt controls compared by t-tests or one-way ANOVA combined with Dunnett's Multiple comparison post hoc test with significance criteria, $* \triangleq p < 0.05,\ ** \triangleq p < 0.01,\ *** \triangleq p < 0.001$.

Supplementary Table 2. Displacement between activated and resting state of individual transmembrane helices (S1 – S4) of the four $Ca_V1.1$ VSDs.

	VSD I / Å	VSD II / Å	VSD III / Å	VSD IV / Å
**S1**	0.6	1.2	3	0.6
**S2**	1.3	1.5	4.8	0.5
**S3**	1.2	1.5	2.5	1.0
**S4**	5.5	3.6	3.9	2.0

Cα-RMSDs of representative activated- and resting state structures of the indicated S1 to S4 helices. We calculated the RMSDs by aligning on the respective VSD. Representative structure the resting state were identified by clustering on the S4 helices of all four VSDs.

Supplementary Table 3. Nucleotide sequences of the primers used to produce the plasmids.

**GFP-$Ca_V1.1$a_VSDI-R4A and GFP-$Ca_V1.1$e_VSDI-R4A**
SalI-F: 5´- cgaaaagagagaccacat -3´
VSDI-R4A-R: 5´- gacaccagcgctagcggtctgagcacacggaaggccttc -3´
VSDI-R4A-F: 5´- ctcagaccgctagcgctggtgtcgggggtgcctagttt -3´
EcoRI-R: 5´- cgtgatccagctcatgta -3´
**GFP-$Ca_V1.1$a_VSDII-K4A and $Ca_V1.1$e_VSDII-K4A**
EcoRI-F: 5´- tcctctactgggtcaacgatg -3´
VSDII-K4A-R: 5´- gctggtgatcgcgaagagcctcaggaggcggatgcagcgcaacacg -3´
VSDII-K4A-F: 5´- tgaggctcttcgcgatcaccagctactggacgtcgctcagcaacct -3´
XhoI-R: 5´- gaacacgcactggaccac -3´
**GFP-$Ca_V1.1$a_VSDIII-R4A and GFP-$Ca_V1.1$e_VSDIII-R4A**
XhoI-F: 5´- ttcactgtggagattgtc -3´
VSDIII-R4A-R: 5´- ttggctccgctgatggctgccaggggccggagcacccttagcac -3´
VSDIII-R4A-F: 5´- cctggcagccatcagcggagccaaagggttgaagcacgtggtcc -3´
BglII-R: 5´- gctggttctcttccagatcttcttgatgatggccctgagctcctc-3´
**GFP-$Ca_V1.1$a_VSDIV-K4A and GFP-$Ca_V1.1$e_VSDIV-K4A**
XhoI-F: 5´- ttcactgtggagattgtc -3´
VSDIV-R4A-R: 5´- tgatcgcgctgctgagtggggccgagggcgtgcgcacgctgctg -3´
VSDII-R4A-F: 5´- tcggccccactcagcagcgcgatcagcctcatgacccggaacag -3´
BglII-R: 5´- gctggttctcttccagatcttcttgatgatggccctgagctcctc-3´
**GFP-$Ca_V1.1$e_VSDII-L_{IV} and GFP-$Ca_V1.1$nc_VSDII-L_{IV}**
EcoRI-F: 5´- tcctctactgggtcaacgatg -3´
VSDII-L_{IV}-R: 5´- cgccacccaggcaatacagtcccccgctggaggccaggaaagtcgactccaccagcagcagctccaggatgcc -3´
VSDII-L_{IV}-F: 5´- tgcctgggtggcggctgcgggaacgttgacccagacgagagcgccggcatctccgtgttgcgctgcatccgcc -3´
XhoI-R: 5´- gaacacgcactggaccac -3´
**GFP-$Ca_V1.1$e_VSDIII-L_{IV} and $Ca_V1.1$nc_VSDIII-L_{IV}**
XhoI-F: 5´- ttcactgtggagattgtc -3´
VSDIII-L_{IV}-R: 5´- ccgcagccgccacccaggcaatacagtcccccgctggaggccaggaaagtggactcgagacccatggagatga -3´
VSDIII-L_{IV}-F: 5´- gtattgcctgggtggcggctgcgggaacgttgacccagacgagagcgccgtggtaaagatcctgagagtgcta -3´
BglII-R: 5´- gctggttctcttccagatcttcttgatgatggccctgagctcctc-3´

Supplementary Figure 1A. Expression and localization in skeletal muscle triads of wildtype and mutant constructs of the adult $Ca_V1.1a$ splice variant in dysgenic myotubes.

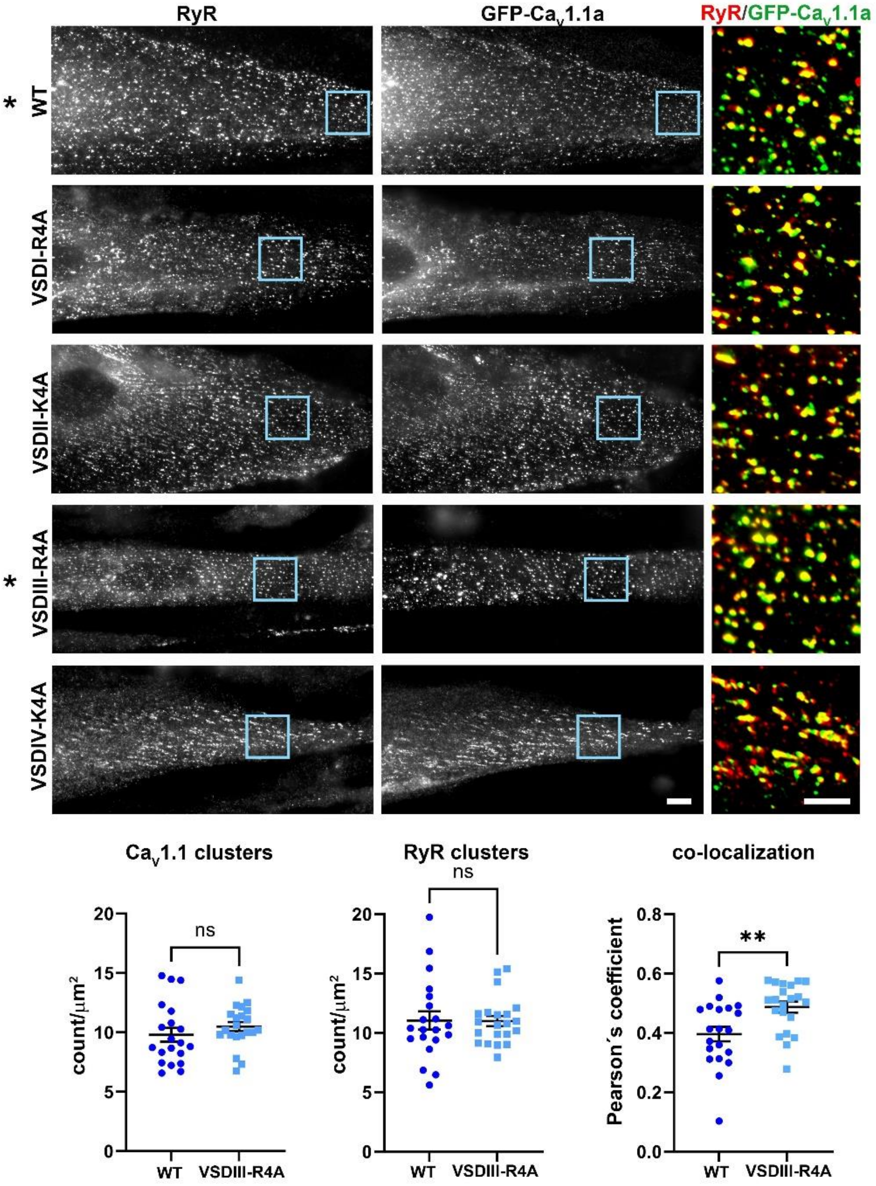

Double-immunofluorescence labeling of the ryanodine receptor (RyR) and $Ca_V1.1$ (anti-GFP) in dysgenic ($Ca_V1.1^{-/-}$) myotubes reconstituted with the adult GFP-$Ca_V1.1a$ splice variant or mutations thereof. Representative myotube of two separate experiments; scale bars, 10 µm. Co-clustering with RyR demonstrates the regular incorporation of the $Ca_V1.1a$ constructs in skeletal muscle triads. The cluster densities and co-localization (Pearson´s coefficient) were compared between wildtype $Ca_V1.1a$ and $Ca_V1.1a$_VSDIII-R4A (asterisks) to examine whether the decreased EC-coupling observed in that mutant (Fig. 3M,O) was due to reduced expression or triad targeting. However, this was not the case. Two-tailed Student T-test, p=0.32 for $Ca_V1.1$ clusters, p=0.95 for RyR clusters and p=0.005 for co-localization.

Supplementary Figure 1B. Expression and localization in skeletal muscle triads of wildtype and mutant constructs of the embryonic $Ca_V1.1e$ splice variant in dysgenic myotubes.

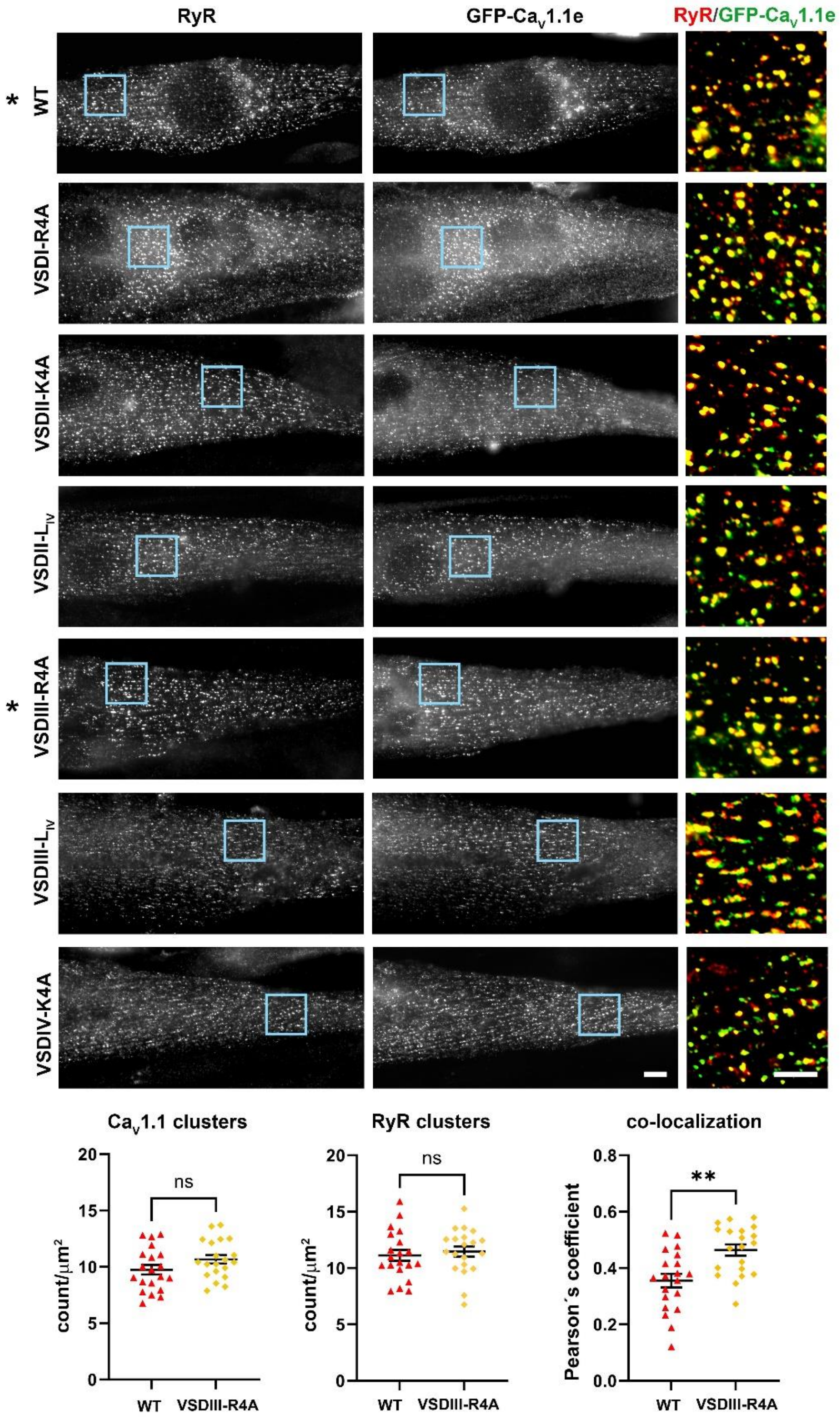

Double-immunofluorescence labeling of the ryanodine receptor (RyR) and $Ca_V1.1$ (anti-GFP) in dysgenic ($Ca_V1.1^{-/-}$) myotubes reconstituted with the embryonic GFP-$Ca_V1.1e$ splice variant or mutations thereof. Representative myotube of two separate experiments; scale bars, 10 µm. Co-clustering with RyR demonstrates the regular incorporation of the $Ca_V1.1e$ constructs in skeletal muscle triads. The cluster densities and co-localization (Pearson´s coefficient) were compared between wildtype $Ca_V1.1e$ and $Ca_V1.1e$_VSDIII-R4A (asterisks) to examine whether the decreased EC-coupling observed in that mutant (Fig. 3M,O) was due to reduced expression or triad targeting. However, this was not the case. (Two-tailed Student T-test, p=0.11 for $Ca_V1.1$ clusters, p=0.61 for RyR clusters and p=0.001 for co-localization).

Supplementary Figure 1C. Expression and localization in skeletal muscle triads of wildtype and mutant constructs of the non-conducting Ca_V1.1nc in dysgenic myotubes.

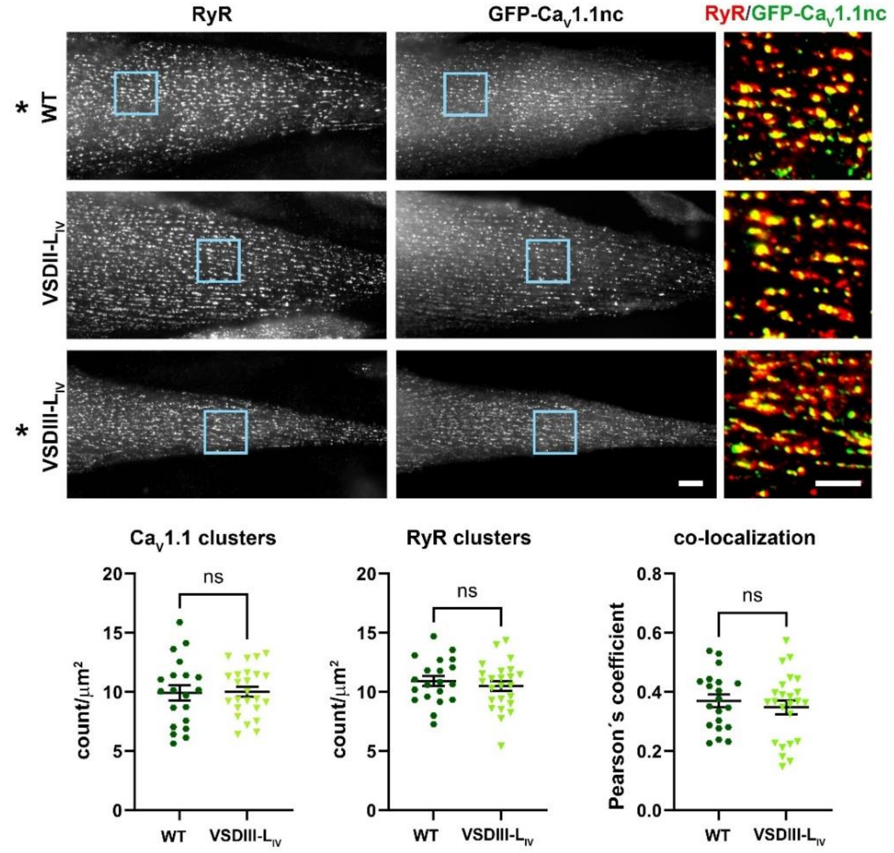

Double-immunofluorescence labeling of the ryanodine receptor (RyR) and Ca_V1.1 (anti-GFP) in dysgenic ($Ca_V1.1^{-/-}$) myotubes reconstituted with the non-conducting GFP-Ca_V1.1nc variant or mutations thereof. Representative myotube of two separate experiments; scale bars, 10 µm. Co-clustering with RyR demonstrates the regular incorporation of the Ca_V1.1nc constructs in skeletal muscle triads. The cluster densities and co-localization (Pearson´s coefficient) were compared between wildtype Ca_V1.1nc and Ca_V1.1nc_VSDIII-L_{IV} (asterisks) to confirm that expression and triad targeting were comparable. Two-tailed Student T-test, $p=0.91$ for Ca_V1.1 clusters, $p=0.46$ for RyR clusters and $p=0.52$ for co-localization.
