# Supplementary material for: CaV1.1 voltage-sensing domain III exclusively controls skeletal muscle excitation-contraction coupling
Source: Nat Commun. 2024 Aug 28;15:7440. doi: 10.1038/s41467-024-51809-5 (PMC11358481; doi:10.1038/s41467-024-51809-5)
Supplement: Supplementary file 3 — Reporting Summary [file 41467_2024_51809_MOESM3_ESM.pdf]

nature portfolio

Corresponding author(s): Bernhard E. FLUCHER

Last updated by author(s): Jul 24, 2024

Reporting Summary

Nature Portfolio wishes to improve the reproducibility of the work that we publish. This form provides structure for consistency and transparency in reporting. For further information on Nature Portfolio policies, see our Editorial Policies and the Editorial Policy Checklist.

Statistics

For all statistical analyses, confirm that the following items are present in the figure legend, table legend, main text, or Methods section.

n/a	Confirmed	
☐	☒	The exact sample size (*n*) for each experimental group/condition, given as a discrete number and unit of measurement
☐	☒	A statement on whether measurements were taken from distinct samples or whether the same sample was measured repeatedly
☐	☒	The statistical test(s) used AND whether they are one- or two-sided<br>*Only common tests should be described solely by name; describe more complex techniques in the Methods section.*
☐	☒	A description of all covariates tested
☒	☐	A description of any assumptions or corrections, such as tests of normality and adjustment for multiple comparisons
☐	☒	A full description of the statistical parameters including central tendency (e.g. means) or other basic estimates (e.g. regression coefficient) AND variation (e.g. standard deviation) or associated estimates of uncertainty (e.g. confidence intervals)
☐	☒	For null hypothesis testing, the test statistic (e.g. *F*, *t*, *r*) with confidence intervals, effect sizes, degrees of freedom and *P* value noted<br>*Give P values as exact values whenever suitable.*
☒	☐	For Bayesian analysis, information on the choice of priors and Markov chain Monte Carlo settings
☒	☐	For hierarchical and complex designs, identification of the appropriate level for tests and full reporting of outcomes
☒	☐	Estimates of effect sizes (e.g. Cohen's *d*, Pearson's *r*), indicating how they were calculated

Our web collection on statistics for biologists contains articles on many of the points above.

Software and code

Policy information about availability of computer code

Data collection	all commercial and open source software used for data collection in this study is specified in the Methods section of the article. No proprietary software has been generated.
Data analysis	all commercial and open source software used for data analysis in this study is specified in the Methods section of the article. No proprietary software has been generated.

For manuscripts utilizing custom algorithms or software that are central to the research but not yet described in published literature, software must be made available to editors and reviewers. We strongly encourage code deposition in a community repository (e.g. GitHub). See the Nature Portfolio guidelines for submitting code & software for further information.

Data

Policy information about availability of data

All manuscripts must include a data availability statement. This statement should provide the following information, where applicable:

- Accession codes, unique identifiers, or web links for publicly available datasets
- A description of any restrictions on data availability
- For clinical datasets or third party data, please ensure that the statement adheres to our policy

The data generated or analyzed during this study are included in this published article (and its extended data). The structures used in this manuscript are publicly

available, with the PDB codes: 5GJW, 6P6W,6N4R respectively. The datasets generated during and/or analysed during the current study are made available alongside the manuscript.

Research involving human participants, their data, or biological material

Policy information about studies with human participants or human data. See also policy information about sex, gender (identity/presentation), and sexual orientation and race, ethnicity and racism.

Reporting on sex and gender	n/a
Reporting on race, ethnicity, or other socially relevant groupings	n/a
Population characteristics	n/a
Recruitment	n/a
Ethics oversight	n/a

Note that full information on the approval of the study protocol must also be provided in the manuscript.

Field-specific reporting

Please select the one below that is the best fit for your research. If you are not sure, read the appropriate sections before making your selection.

☒ Life sciences ☐ Behavioural & social sciences ☐ Ecological, evolutionary & environmental sciences

For a reference copy of the document with all sections, see nature.com/documents/nr-reporting-summary-flat.pdf

Life sciences study design

All studies must disclose on these points even when the disclosure is negative.

Sample size	between 5 and 12 (typically >8), duplicated in two channel variants (makes a minimum of 13 recordings for each experimental condition)
Data exclusions	all recordings are checked for pre-defined quality criteria for inclusion in analysis;
Replication	multiple samples from at least 3 indecpendent experiments (i.e. separate cell passages and transfections)<br>all experiments performed in two different channel splice variants; all experiments were replicable
Randomization	all experimental conditions (i.e. channel mutants and variants) are compared with matched controls (WT) form the same experiment; sometimes several experimental conditions are recorded in the same set of experiments
Blinding	n/a because the characteristic current / calcium transient properties reveal the identity of experimental condition and controls

Reporting for specific materials, systems and methods

We require information from authors about some types of materials, experimental systems and methods used in many studies. Here, indicate whether each material, system or method listed is relevant to your study. If you are not sure if a list item applies to your research, read the appropriate section before selecting a response.

Materials & experimental systems

n/a	Involved in the study	
☐	☒	Antibodies
☐	☒	Eukaryotic cell lines
☒	☐	Palaeontology and archaeology
☒	☐	Animals and other organisms
☒	☐	Clinical data
☒	☐	Dual use research of concern
☒	☐	Plants

Methods

n/a	Involved in the study	
☒	☐	ChIP-seq
☒	☐	Flow cytometry
☒	☐	MRI-based neuroimaging

Antibodies

Antibodies used	polyclonal rabbit anti-GFP antibody (serum, 1:10,000; A6455 Thermofisher Scientific); monoclonal mouse anti-RyR (34C, 1:500; MA3-925 Thermofisher Scientific); fluorescently labeled with Alexa-488 and Alexa-594
Validation	controls: non-transfected cells; non-muscle cells; omission of primary ABs

Eukaryotic cell lines

Policy information about cell lines and Sex and Gender in Research

Cell line source(s)	GLT myotubes: immortalized CaV1.1-null mouse myoblasts (Powell et al., 1996; https://doi.org/10.1083/jcb.134.2.375)
Authentication	cell line continuously maintained in the author´s lab since its generation in 1995
Mycoplasma contamination	n/a because fresh cells are thawed out after every 20-25 passages and quality of cultures (e.g. myotube differentiation) is continuously monitored
Commonly misidentified lines (See ICLAC register)	n/a

Plants

Seed stocks	n/a
Novel plant genotypes	*Describe the methods by which all novel plant genotypes were produced. This includes those generated by transgenic approaches, gene editing, chemical/radiation-based mutagenesis and hybridization. For transgenic lines, describe the transformation method, the number of independent lines analyzed and the generation upon which experiments were performed. For gene-edited lines, describe the editor used, the endogenous sequence targeted for editing, the targeting guide RNA sequence (if applicable) and how the editor was applied.*
Authentication	*Describe any authentication procedures for each seed stock used or novel genotype generated. Describe any experiments used to assess the effect of a mutation and, where applicable, how potential secondary effects (e.g. second site T-DNA insertions, mosiacism, off-target gene editing) were examined.*
